# Exploiting Manipulated Small Extracellular Vesicles to Subvert Immunosuppression at the Tumor Microenvironment through Mannose Receptor/CD206 Targeting

**DOI:** 10.3390/ijms21176318

**Published:** 2020-08-31

**Authors:** Maria Luisa Fiani, Valeria Barreca, Massimo Sargiacomo, Flavia Ferrantelli, Francesco Manfredi, Maurizio Federico

**Affiliations:** National Center for Global Health, Istituto Superiore di Sanità, 00161 Rome, Italy; barrecavaleria@gmail.com (V.B.); massimo.sargiacomo@iss.it (M.S.); flavia.ferrantelli@iss.it (F.F.); francesco.manfredi@iss.it (F.M.)

**Keywords:** tumor-associated macrophages, tumor microenvironment, macrophage polarization, mannose receptor, exosomes, extracellular vesicles, HIV-1 Nef

## Abstract

Immunosuppression at tumor microenvironment (TME) is one of the major obstacles to be overcome for an effective therapeutic intervention against solid tumors. Tumor-associated macrophages (TAMs) comprise a sub-population that plays multiple pro-tumoral roles in tumor development including general immunosuppression, which can be identified in terms of high expression of mannose receptor (MR or CD206). Immunosuppressive TAMs, like other macrophage sub-populations, display functional plasticity that allows them to be re-programmed to inflammatory macrophages. In order to mitigate immunosuppression at the TME, several efforts are ongoing to effectively re-educate pro-tumoral TAMs. Extracellular vesicles (EVs), released by both normal and tumor cells types, are emerging as key mediators of the cell to cell communication and have been shown to have a role in the modulation of immune responses in the TME. Recent studies demonstrated the enrichment of high mannose glycans on the surface of small EVs (sEVs), a subtype of EVs of endosomal origin of 30–150 nm in diameter. This characteristic renders sEVs an ideal tool for the delivery of therapeutic molecules into MR/CD206-expressing TAMs. In this review, we report the most recent literature data highlighting the critical role of TAMs in tumor development, as well as the experimental evidences that has emerged from the biochemical characterization of sEV membranes. In addition, we propose an original way to target immunosuppressive TAMs at the TME by endogenously engineered sEVs for a new therapeutic approach against solid tumors.

## 1. Introduction

Both immunosuppression and genetic escape are formidable weapons through which tumors can elude host immune surveillance. Solid tumors develop in a quite complex context, referred to as tumor microenvironment (TME) [1,2], which is composed of both cellular and non-cellular elements, usually resulting in an immunosuppressive behavior. Counteracting such a general effect would favor both spontaneous and therapeutic anti-tumor immunity, hence critically contributing to control tumor cell growth. Therefore, subverting TME immunosuppression represents a major goal for anticancer immunotherapies.

**Figure 1:**
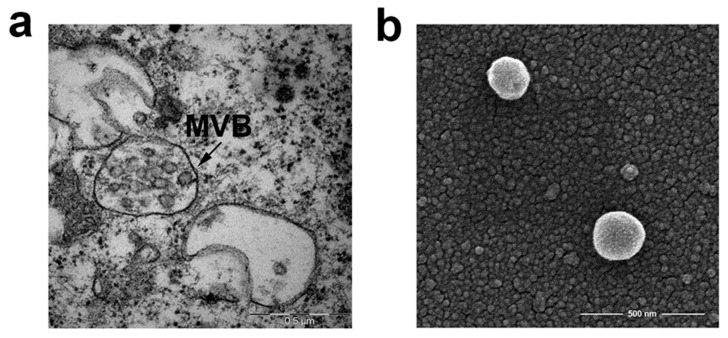
Electron microscopy of multivesicular bodies (MVB) and small extracellular vesicles (sEVs) (**a**) TEM micrograph of multivesicular bodies with intraluminal vesicles in Mel501, a melanoma cell line (**b**) SEM (Scanning Electron Microscope). Micrograph of sEVs purified from conditioned medium of Mel501 cells by differential centrifugations. Courtesy of Francesca Iosi and the Microscopy Area of the ISS Core Facilities.

Both normal and tumor cells constitutively release membrane-bilayered vesicles, commonly referred to as extracellular vesicles (EVs) [3,4]. They differ in the mechanisms of biogenesis and secretion, giving rise to the generation of a heterogeneous population of vesicles with different sizes and contents [5,6], which include small EVs (sEVs) or exosomes and microvesicles or ectosomes. Exosomes are vesicles of 30–150 nm diameter generated by inward budding of endosomal membranes to form intraluminal vesicles that accumulate in intracellular organelles called multivesicular bodies (MVBs). MVBs ultimately fuse with the plasma membrane, thereby releasing intraluminal vesicles into the extracellular environment (Figure 1). On the contrary, ectosomes are 100–500 nm vesicles shed by direct budding from the plasma membrane [7,8]. Different types of EVs often show overlapping features that make difficult to obtain relatively pure preparations when purified from cell-conditioned media or biological fluids. In this review, we will use the term sEV to refer to EV types co-isolated by typical purification methods and exosomes to distinguish EV whose subcellular biogenesis strictly derives from multivesicular bodies/endosomes [8].

SEVs carry a complex cargo of nucleic acids, proteins, and lipids that largely reflects the characteristics and the functional state of the cells they originate from, and that will be delivered to neighboring or distant cells [9,10]. As a result, the functions of those recipient cells will be modulated by sEVs in a manner that is strictly dependent on the nature of producer cells, making sEVs central players in intercellular communication and reprogramming of target cells [11]. Ectosomes generation is a much less known process that requires the accumulation of their cargo at the cytosolic surface of specific plasma membrane microdomains [7,12].

SEVs-mediated transfer of molecular and genetic material from one cell to another, either locally or at long distance, is a key contributor to the mechanisms of intercellular communication involved in various physiological and pathological conditions [13,14,15]. Moreover, for these reasons, sEVs are now considered powerful tools for clinical applications, including advanced diagnostics, therapeutics, and regenerative medicine [16,17,18,19].

The molecular composition of sEVs is determined by the cell type of origin as well as by the intracellular pathway followed en route to their release into the extracellular space [8,20,21]. This heterogeneity confers to sEVs distinct properties, such as tropism to certain organs, and uptake by specific cell types. In the case of tumor-derived sEVs, these events often lead to the impairment of immune responses at TME [22], also favoring pre-metastatic niche formation and metastasis [23,24]. 

In tumor cells, sEV biogenesis and ultimately sEV composition is a complex and regulated process, which involves many different molecules associated with the sEV biogenesis pathway [4,25]. Whatever the cell type of origin, sEVs can be characterized, although not exclusively, by the presence of different types of cell surface proteins, such as tetraspanins, (i.e., CD9, CD81, CD63), ESCRT (endosomal sorting complex required for transport) proteins (Alix and TSG101), integrins, RNA, DNA, lipids such as ceramide and the atypical phospholipid, lysobisphosphatidic acid (LBPA) [26], and oligosaccharides [27].

TME exerts a key influence on tumor cells, and the resulting sEVs, responsible for proteins and genetic material transfer from primary tumor cells, play a crucial role in metastatic colonization and in the formation of the pre-metastatic niche, driving recipient cells to acquire a pro-tumorigenic phenotype [23,28]. The selective conditions present in TME, such as the generation of a hypoxic [29] and acidic environment [30], strongly influence sEV secretion by tumor cells, thus contributing to the malignant tumor phenotype. Furthermore, sEV membrane composition reflects TME changes and conceivably influence and control the different mechanisms of entry or interaction of sEVs with target cells supporting tumor growth [31,32].

Interestingly, it has been described that major players of immunosuppression at the TME, i.e., immunosuppressive tumor-associated macrophages (TAMs), express on their surface high levels of mannose receptor (MR, CD206) [33]. The MR is an endocytic receptor with a high affinity for high mannose oligosaccharides, glycans highly enriched on the surface of sEVs [34]. In this review, literature data regarding both TAM functions and the molecular structure of sEVs are reviewed. In addition, we propose an original way to exploit typical molecular signatures of both TAMs and sEVs to counteract the immunosuppression at the TME.

## 2. The Tumor Microenvironment

In solid tumors, cancer cells are embedded within a milieu that favors their proliferation and comprises both cellular and non-cellular components. Fibroblasts, endothelial cells, and essentially all types of immune cells are part of the TME [35,36]. Among non-cellular components, tumor-derived sEVs play a key role in immune suppression. TME composition can vary among different tumors, and between primary and metastatic neo-formations in the same patient, and is tightly associated with the clinical outcome of cancer patients.

TMEs can be categorized based on different criteria. In terms of abundance of tumor-infiltrating cytotoxic CD8+ T lymphocytes (CTLs), TMEs can be distinguished in either hot/inflamed, with the highest content of CTLs, or cold/desert, with a virtual absence of infiltrated CTLs [37]. TME core infiltrated by CTLs represents a favorable condition for an effective anticancer immune response, both spontaneous and induced by immunotherapeutic interventions.

**Figure 2:**
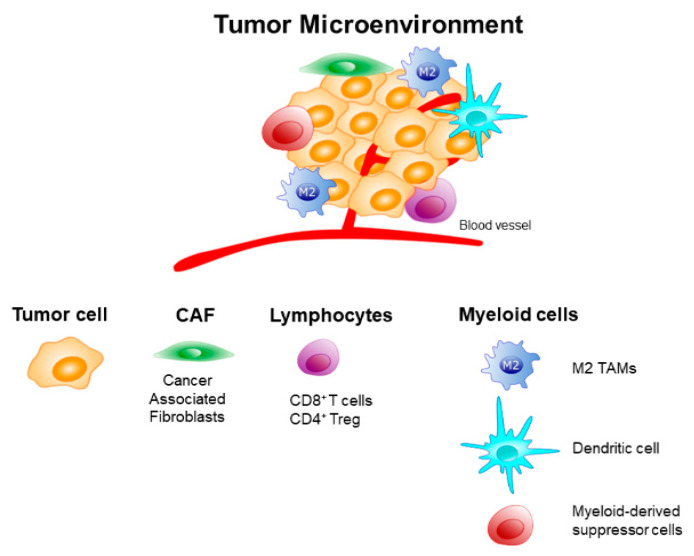
Schematic representation of cells populating the tumor microenvironment (TME).

TME is populated by different kinds of immune cells having immune suppressive actions. Among these are myeloid-derived suppressor cells, neutrophils, CD4+ Treg lymphocytes, and immunosuppressive M2-like TAMs [38]. These latter cells can represent up to 50% of the tumor mass, and play a key role in the immune evasion at TME by secreting proteases, angiogenic factors, and pro-tumoral products. The functional plasticity of TAMs modifies their phenotype and activity in response to a great number of microenvironmental stimuli, although the mechanisms that determine the different polarization states are still to be elucidated [39]. These different functional states often coexist and can significantly vary between different tumors [40,41]. TAMs can also dispose at the tumor margin, where they can interact with CTLs, thus inhibiting their infiltration towards tumor cells [42]. For all these reasons, immunosuppressive TAMs have been identified as a major cell target for novel designs of cancer immunotherapies focused on improving the overall anti-tumor immune response. A schematic representation of cells populating TEM is illustrated in Figure 2.

## 3. The TAM-Mediated Immunosuppression at the TME

A large number of macrophages infiltrate solid tumors, thereby influencing several aspects of tumor development [43]. The most relevant effects include suppression of anticancer immunity, angiogenesis promotion, and support for metastasis. Macrophages are recruited at TME, in response to the secretion by tumor cells and other TME cell types, of a number of chemoattractant soluble factors, including vascular-endothelial growth factor A (VEGF-A) [44], chemokine ligand 2 (CCL2) [45], and colony-stimulating factor 1 (CSF-1) [46].

TME-populating macrophages can be schematically distinguished in M1- and M2-like macrophages. M1-like macrophages show both pro-inflammatory and immune-stimulatory properties, thus exerting an anti-tumor function. On the other hand, M2-like macrophages favor tumor angiogenesis and immunosuppression. Such a distinction, although useful from both therapeutic and diagnostic points of view, is now outdated, due to the identification of a large number of intermediate subclasses, i.e., up to 19 [47]. They have been identified through most recent transcriptomic techniques, e.g., single-cell mass cytometry and single-cell RNA sequencing [48,49,50], and in vivo represent a continuum of functional phenotypes with intermediates showing overlapping features.

TAMs can be characterized by the expression of different surface markers [47,51], distinct metabolic changes [52,53], and a broad transcriptional repertoire with the involvement of key transcription factors, which can be activated by the environmental signals received. In particular, members of the signal transducer and activator of transcription (STAT), peroxisome proliferator-activated receptors (PPARs), interferon regulatory factor (IRF), and nuclear transcription factor-κB (NF-κB) families are essential for macrophage polarization toward the M1 profile [41,54,55].

M2-like TAMs contribute to tumor angiogenesis by secreting soluble factors inducing endothelial cell proliferation, including VEGF-A, interleukin (IL)-1β, IL-6, tumor necrosis factor (TNF)α, CXCL8, and fibroblast growth factor (FGF)-2 [56]. In particular, the secretion of VEGF-A by perivascular TAMs can increase vascular permeability and access of tumor cells to peripheral blood circulation [57]. On the other hand, the production of proteases, e.g., matrix-metalloproteases, induces degradation of extracellular matrix and the consequent liberation of embedded soluble factors released by both cancer and stromal cells having pro-tumoral effects and favoring metastasization.

**Figure 3:**
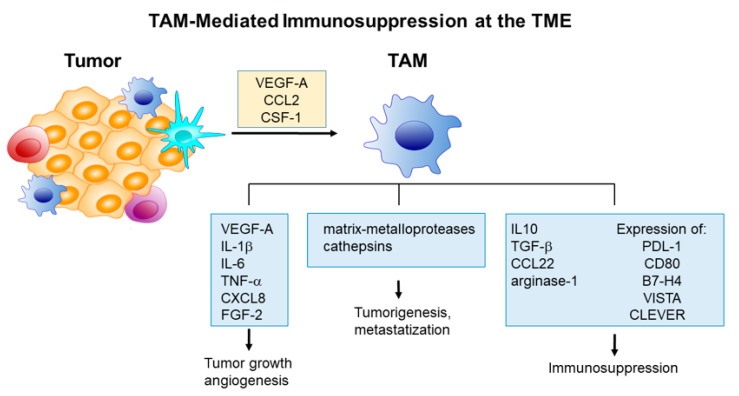
Principal mechanisms of tumor-associated macrophage (TAM) mediated immunosuppression at the tumor microenvironment (TME).

TAM-mediated immunosuppression at TME is essentially mediated by three concurrent mechanisms: (i) Release of soluble immunosuppressive factors, e.g., IL-10, CCL22, and transforming growth factor (TGF)-α as well as factors recruiting regulatory T cells (Treg) [58]; (ii) expression of ligands for lymphocyte suppressor factors PD-1 and CTLA-4, i.e., PDL-1 and CD80, as well as other checkpoint inhibitors with similar functions, including B7-H4, V-domain Ig suppressor of T cell activation (VISTA) [59], and vascular endothelial receptor (CLEVER) [60], and (iii) starving the TME of l-arginine, i.e., an essential factor for T-cell activity, through the release of arginase-1 [61]. Figure 3 illustrates the principal mechanisms of TAM mediated immunosuppression at the TME.

The multiple immunosuppressive signals at play within the TME greatly reduce the efficacy of current immunotherapies. Therefore, new strategies to effectively reprogram the various immunosuppressive cell types at the TME are urgently needed.

## 4. Re-Programming of TAMs

**Figure 4:**
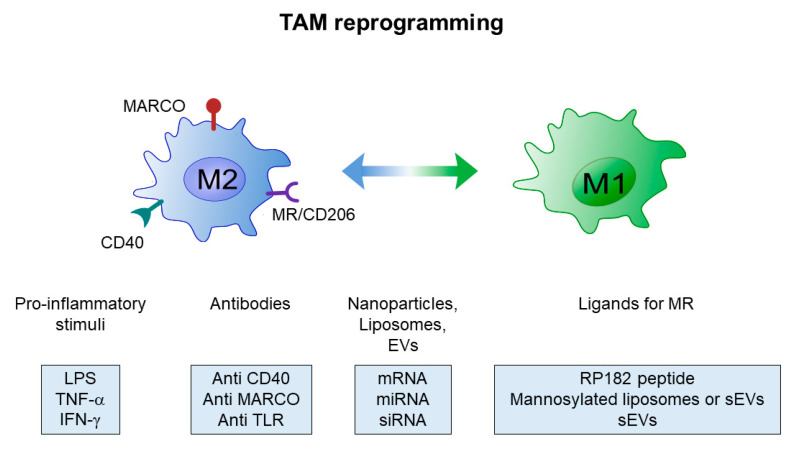
Scheme of current strategies for tumor-associated macrophage (TAM) reprogramming.

Immunosuppressive TAMs represent a privileged therapeutic target for the treatment of solid tumors, especially in the case immune checkpoint blockers (ICBs) are used. Given the enormous therapeutic value of TAMs re-education towards M1-like macrophages to promote tumor regression, much attention has focused on effective strategies aimed at targeting TAMs, including the blockade of the M2 phenotype, enhanced activation of M1 macrophages and reprogramming of TAMs toward M1-like phenotype [58,62,63,64,65]. Many of these different approaches against immunosuppressive TAMs have been summarized in Table 1 and strategies directed at TAM reprogramming illustrated in Figure 4.

Promising results have been obtained with direct activation of M1-like macrophages by Th1 cytokines like IFN-γ [87], and by targeting toll-like receptors (TLR) and/or CD40 with agonists and monoclonal antibodies [97,98,99]. However, the onset of systemic inflammation limited the therapeutic efficacy of these approaches in vivo, and additional investigations are ongoing to circumvent this hurdle. In any case, considering the functional plasticity of macrophages, re-educating M2- versus an M1-like macrophage phenotype currently appears the most attractive therapeutic option.

A major hindrance to effective targeting of immunosuppressive TAMs is represented by the scarcity of specific protein markers expressed on M2 macrophages. Some potential targets, whose expression also correlates with poor prognosis, have been investigated. Among them, the selective targeting of MARCO (macrophage receptor with collagenous structure) with monoclonal antibodies has been recently used to promote a switch to an M1-activated phenotype [100].

Increasing evidence suggests that a valuable alternative is represented by targeting the MR, which is highly expressed on M2, but not M1 macrophages [92,103,104,105].

## 5. The Mannose Receptor in M1 Polarization

MR is expressed on TAMs where is a prototypical marker of M2-type activation. It is also expressed on the surface of immature dendritic cells (DCs), liver sinusoidal endothelial cells, and other tissue macrophages. Earlier studies have demonstrated that MR expression is strongly down-regulated by IFN-γ [106], and upregulated by interleukin-4 (IL-4) [107]. The MR is a 175 kDa Type I integral membrane protein that belongs to the family of C-type lectin receptors and binds glycoconjugates terminated in mannose, fucose, or *N*-acetil-β-d-glucosamine (GlcNAc) in a calcium-dependent manner [108,109,110]. The receptor contains three distinct extracellular domains, i.e., an N-terminal cysteine-rich domain (CR) that binds sulfated carbohydrates, a fibronectin type II domain (FNII) that binds collagen, and eight tandem C-type lectin carbohydrate-recognition domains (CRDs) [111,112]. CRDs have only weak affinity affinities for single sugars, and several CRDs need to be clustered to achieve high-affinity binding to oligosaccharides. This clustering allows for the internalization of mannosylated proteins and other exogenous molecules, including allergens and microbial products.

MR is a highly effective clathrin-dependent endocytic receptor that constantly recycles between the plasma membrane and the early endosomal compartment [111]. Most part of MRs is intracellular, while only ∼15% of the cellular pool can be found on the cell surface. Like other members of the C-type lectin receptor family, the MR undergoes conformational changes upon ligand binding or as pH decreases in intracellular compartments [111,113]. Once acidification takes place in the endosomal compartment, the MR dissociates from its ligands, and the empty receptor recycles back to the plasma membrane.

Several approaches have been adopted to target the MR and selectively deliver therapeutic nanoparticles. Among these, drug-free mannosylated liposomes have been shown to induce effective anti-tumor activity by enhancing the expression ratio of CD86/MR [102]. In another study, mannosylated nanoparticles suitable for intracellular delivery of drug carriers have been shown to selectively target with high specificity MR expressing macrophages [104].

MR conformational changes that occur upon ligand binding have been recently exploited to target M2 macrophages and induce reprogramming toward M1 phenotypes. For instance, precision targeting with short peptides showed some potential for the intracellular delivery of therapeutically relevant molecules [114]. In addition, a very recent report showed that direct binding of MR with a synthetic peptide (RP-182), i.e., an analogue of naturally occurring antimicrobial peptides, activates phagocytosis and autophagy in M2-like macrophages, reverting these cells into an anti-tumor M1-like phenotype with increased M1 cytokine production and phagocytosis of cancer cells [103].

On the other hand, also cell-secreted sEVs can be considered attractive candidates to specifically target M2-like macrophages via the MR, since they expose high mannose and other classes of N-linked oligosaccharides on their surface [115,116].

## 6. Extracellular Vesicles for Anti-Tumor Therapy

Pioneering studies have shown that sEVs secreted by DCs pulsed with cancer peptides successfully eradicate established tumors in mice [117]. Furthermore, tumor-derived EVs are a source of neoantigens that, once internalized by DCs, could cross-prime CD8+ T cells and lead to tumor rejection [118]. Since these early studies, the field of sEVs-based cancer therapeutics has attracted many efforts, and sEVs have emerged as promising tools for targeted drug delivery. Despite a growing interest in these nanovesicles as natural carriers, there are still many open questions that need further investigation. For example, specific recognition by target cells is of fundamental importance for an effective delivery of bioactive molecules. EVs uptake may occur via receptor-mediated endocytosis or phagocytosis, or direct fusion with the plasma membrane. Some studies have pointed at integrins [24] and scavenging receptors [119] as mediators of EVs targeting, but current knowledge on this matter is rapidly evolving and has been recently comprehensively reviewed [5,7,120,121,122].

**Figure 5:**
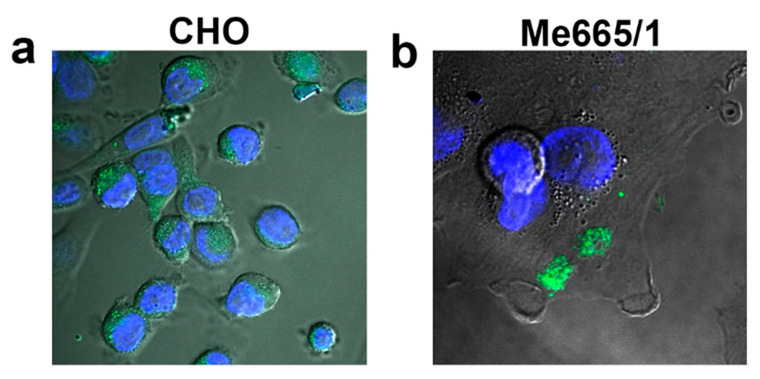
Fluorescent sEVs uptake by different cell lines. Confocal fluorescence microscopy images of green fluorescent sEVs derived from melanoma Me665/1 cells transferred on (**a**) CHO cells and (**b**) Me665/1 cells in nonspecific conditions [66]. Blue-fluorescent nuclei are stained with DAPI.

The different modes of sEV uptake may result in distinct localization and functional effects of the sEVs components, but it is still unknown whether a specific route of entry is to be preferred for a successful transfer of EVs cargo. Thus, understanding through which mechanisms sEVs deliver their content into target cells is a central point that needs to be further elucidated. To the best of current knowledge, while either non-selective uptake or direct fusion with the plasma membrane of target cells seem to be the preferential mechanisms of bulk sEV incorporation (Figure 5) [123], alternative and more specific routes of uptake may depend on the characteristics of surface components of both sEV and target cells.

Tumor-derived EVs can be efficiently taken up by DCs for antigen processing and cross-presentation to tumor-specific CTLs [124]. Immature DCs (iDCs) internalize EVs more efficiently than mature DCs, whereas mature DCs retain more EVs on the cell surface [125]. The surface of iDCs harbors sugar-binding C-type lectin receptors (CLRs) [126,127], which is a characteristic shared with M2-like macrophages.

Glycomic studies conducted to date demonstrate that surface glycoprofiles of sEVs contain high amounts of mannose and other classes of N-linked oligosaccharides [27,34,128,129] making them suitable ligands for the MR. Recently, it has also been shown that mannose-modified serum sEVs display elevated uptake by murine DCs [116]. This evidence, together with a number of studies showing that mannosylation of both liposomes [102] and synthetic nanocarriers [130,131] enhance cellular uptake by M2 macrophages, inducing stimulation and polarization of macrophages toward the M1 phenotype, point to sEVs as powerful ligands of M2 macrophages. Furthermore, the affinity of EVs membrane components (i.e., proteins, lipids, and glycans) for certain tissues greatly affects biodistribution of EVs in vivo, thus, encouraging studies aimed at altering the surface of EVs to improve targeting to selected organs. Many different strategies have been adopted, but there is now accumulating evidence that carbohydrates on the vesicle surface participate in the recognition and uptake of EVs by phagocytes. Interestingly, manipulation of surface glycans on EVs either by removal of sialic acid [132] or by treatment with *N*-glycosidases [133,134] alters the uptake capacity of different cells. In the first case, the change also affects the in vivo biodistribution of sEVs showing accumulation of desialylated sEVs in the lungs. Altogether, these results point to the importance of *N*-glycosylation in cellular uptake, but since different cell types respond differently to glycosylation changes appears evident that the cell to be targeted, with its endowment of specific protein receptors, represent the cornerstone of receptor ligand recognition.

## 7. Molecular Basis of TAM Re-Programming by Engineered sEVs

The subversion of TAM-mediated immunosuppression at TME through macrophage transcriptional reprogramming represents a quite attractive option for anticancer combined immunotherapy. Like normal cells, tumor cells also constitutively release sEVs, which interact primarily with TME cell constituents. SEVs, as naturally occurring vesicles, have a low intrinsic immunogenic profile, are able to avoid, at least in part, the degradative pathway and possess the ability to overcome the blood-brain barrier. Consequently, they have emerged as an important means to deliver therapeutic agents. In this context, engineering tumor-derived sEVs with molecules inducing an inflammatory-like macrophage phenotype would be instrumental in alleviating the immunosuppression at TME. Over the past few years, several engineering strategies have been devised to manipulate tumor-derived sEVs in order to induce cellular and innate immunity. SEV engineering can be carried out either at the level of producer cells or directly on purified sEVs. The different approaches used mostly depend on cargo properties, such as hydrophilicity, hydrophobicity, and molecular weight, as each method has a different loading capacity. For example, chemicophysical methods like electroporation are widely used for loading relatively large molecules. such as siRNA or miRNA into sEVs [19]. Particularly for cancer research, the observation that exosomal miRNAs effectively engage target mRNA and suppress gene expression in recipient cells has been heavily favored. Due to the availability of various cellular engineering methods, different types of RNAs that are released via sEVs have also been exploited and recently reviewed [11].

**Figure 6:**
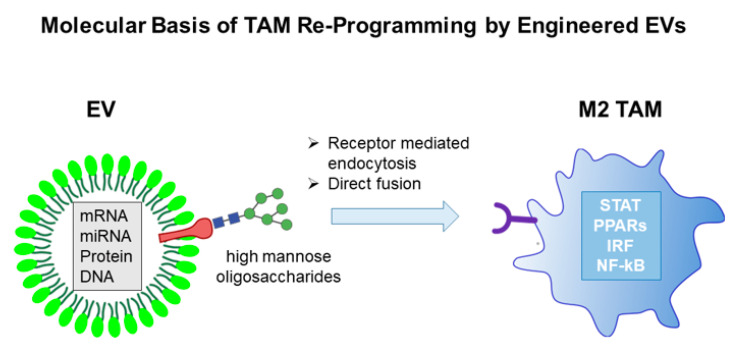
TAMs reprogramming by engineered EVs.

During the past decade, many efforts have been devoted to transfect sEV-producing cells with plasmids encoding protein sequences that, once uploaded in the nanovesicles, are able to alter the phenotype of target cells [135,136,137,138]. However, a method to target M2 macrophages with sEVs capable of inducing their reprogramming to M1 anti-tumor phenotype is not yet available. One major hurdle in EVs research for an effective therapeutic application is represented by the lack of optimized isolation, characterization and quantification procedures. Various purification techniques, such as differential ultracentrifugation, density gradients, precipitation, filtration, size exclusion chromatography, and immunoisolation are currently used to obtain less heterogeneous sEVs preparation; however, quantification often relies on the total protein content of EVs. Fluorescent labeling techniques are more accurate and have the main advantage that track EVs in vivo [139,140]. The different approaches for TAMs reprogramming by engineered EVs are schematically illustrated in Figure 6.

## 8. HIV-1 Nef Protein as Effector of TAM Reprogramming

A plausible candidate for reprogramming M2 macrophages is the Human immunodeficiency virus (HIV)-1 Negative Regulatory Factor (Nef) protein [141], a 27 kilodalton (kDa) scaffold protein, which lacks enzymatic activities. After synthesis at free ribosomes, Nef reaches both intracellular and plasma membranes with which it tightly interacts through its N-terminal myristoylation. Nef acts as a scaffold/adaptor element in triggering activation of signal transducing molecules like p21 PAK-2, NF-κB, STATs, ERK1/2, Vav, and Src family kinases. In most cases, signal activation occurs upon Nef association with lipid raft microdomains at cell membranes [142,143,144]. The fact that also sEV membranes are enriched in lipid raft microdomains explains why Nef can be found in EVs [145,146,147,148,149]. 

Cumulate literature data demonstrate that the presence of Nef inside macrophages induces a strong pro-inflammatory response. In particular, Nef switches on the transcription of many inflammatory genes, as well as the release of inflammatory factors like CCL3, CCL4, IL-1β, IL-6, TNF-α [150,151], and interferon gamma (IFN)-γ [152]. This potent pro-inflammatory response is mediated by the activation of several signal transduction molecules, including STAT-1, 2, and 3, NF-κB, JNK, ERK1/2, and MAPK [153,154]. The inflammatory effects of Nef on macrophages depends on four glutamate-acidic cluster domain located at 62–65 amino acid position [155].

Data from many independent investigation groups strongly support the idea that Nef associates with EVs at low levels [156,157]. Conversely, we identified a Nef mutant incorporating in sEVs/EVs at quite high levels [158]. This Nef mutant (referred to as Nef^mut^) is defective for the most part of Nef functions, including down-regulation of cell membrane receptors, Nef-associated kinase (NAK) activation, an increase of HIV expression [159]. Nevertheless, it maintains an unaltered acidic cluster domain that correlates with the induction of cell activation in antigen-presenting cells when it is delivered by nanovesicles [160].

**Figure 7:**
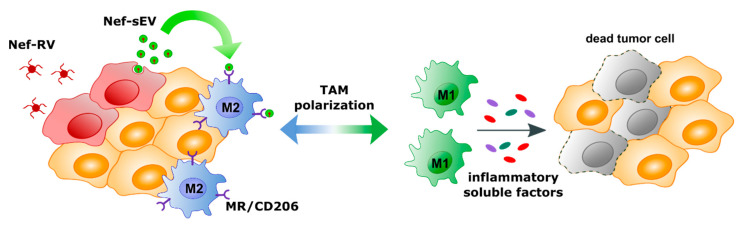
Scheme of the proposed mechanism for TAM reprogramming. Tumor cells are transfected with retroviral vectors expressing Nef^mut^ (Nef-RV). Nef-engineered sEVs (Nef-sEV) are then released into the TME infiltrated with M2 like macrophages expressing the mannose receptor (MR/CD206). MR mediated uptake of Nef-Sev might induce polarization of M2 into M1 like macrophages—ultimately leading to the release of pro-inflammatory factors.

Considering this evidence, one may hypothesize that the delivery of Nef^mut^-engineered EVs inside M2-TAMs would be instrumental in re-educating macrophages at TME from an M2-like to an M1-like phenotype. In the case of solid tumors, this design could be applied through a quite simple strategy, i.e., tumor cell engineering for Nef^mut^ expression through retroviral vector-mediated transduction. In this way, only actively replicating cells at the TME, hence preferably tumor cells, are expected to be transduced. Nef-engineered tumor-derived sEVs may diffuse within TME, thereby preferentially entering MR positive TAMs in view of the mannose expressed on the sEV surface. Once internalized by macrophages, Nef^mut^ might switch on intracellular signals—ultimately leading to the release of pro-inflammatory factors. These factors might act in an autocrine/paracrine loop to induce the reprogramming of macrophage transcriptional profile toward the M1-like phenotype (Figure 7).

Eventually, this mechanism, which essentially hijacks the sEV-mediated intercellular communication at the TME, is expected to alleviate the immune suppression at the TME, thereby favoring the action of anticancer adaptive immune responses.

Hopefully, once supported by experimental confirmation, this design would have a therapeutic utility in the battle against solid tumors.

## 9. Conclusions

Immunosuppression at TME protects cancer cells from both spontaneous and artificially generated host immune responses. Hence, subverting immunosuppression should be considered a priority for any anticancer immunotherapeutic strategy. Even if M2-like TAMs are major players, other cell types contribute to the general immunosuppression at TME, including CD4+ Tregs lymphocytes, myeloid-derived suppressor cells, and neutrophils [42]. Similar to macrophages, neutrophils can polarize in pro- and anti-tumor phenotypes, depending on the stimuli they receive at TME. Interestingly, it has been reported that IFN-γ can polarize neutrophils towards an anti-tumor phenotype [161]. Considering the quite high levels of IFN-γ transcripts induced by Nef in macrophages, the delivery of Nef^mut^-engineered, tumor-derived sEVs to M2-like TAMs is expected to have paracrine anti-tumor effects also on neutrophils that populate the TME.

The strategy we propose is certainly only one of many potential new anti-tumor therapeutic approaches that the manipulation of sEVs/EVs can offer. The increase in knowledge of the sEV/EVs biology, mainly regarding the mechanisms of cell entry, will favor the implementation of new and more efficient therapeutic approaches against tumors and infectious diseases.

## Figures and Tables

**Table 1:** Selected strategies to target tumor-associated macrophages (TAMs).

Mechanism of Action	Active Agent	Vehicle Carrier	Target	References
Depletion of M2 TAMs	Shiga toxins	Shigella Flexneri attenuated strain	TAMs	[66]
	Immunotoxins		TAMs Receptors	[67,68]
	Bisphosphonates (e.g., clodrolip, zoledronic acid)	Liposomes	TAMs, Kupffer cells	[69,70]
	Trabectedin		TAMs	[71]
	Tyrosine Kinase Inhibitors (e.g., Dasatinib, Bosutinib)		endothelial and myeloid cells in TEM, TAMs	[72,73]
Inhibition of circulating monocyte recruitment into tumor	CCR2 inhibitors; anti-CCR2/CCL2 blocking antibodies		TAMs CCR2	[45,74,75,76]
	Antagonists of CXCL12/CXCR4 axis		TAMs CXCR4	[77,78]
	anti-CSF-1R antibody		TAMs CSF-1R	[79,80]
	neutralizing CD11b antibody		CD11b on Myeloid Cells	[81,82]
Blockade of M2 Phenotype	Tyrosine kinase inhibitors or drugs blocking STAT3		TAMs STAT3	[83,84]
	drugs blocking STAT6		TAMs STAT6	[85]
Enhanced Activation of M1 Macrophages	Th1 cytokines like IFN-γ		TAMs STAT1 stimulation	[86,87]
	metformin		TAMs AMPKα1 stimulation	[88]
	toll-like receptor agonists, CpG-ODNs; PI3Kγ deletion		TAMs NF-κB stimulation	[89,90,91]
Reprogramming TAMs Toward M1-Like Phenotype	mRNAs; miRNA	Targeted Nanocarriers	TAMs	[92,93]
	siRNA	Different types of Nanoparticles	TAMs	[94,95,96]
	anti-CD40 antibody		TAMs CD40	[97,98,99]
	anti-MARCO antibody		TAMs MARCO	[100]
	gefitinib/vorinostat	Trastuzumab-modified Mannosylated Liposomes	TAMs MR	[101]
	Drug free	Mannosylated Liposomes	TAMs MR	[102]
	RP-182 Peptide		TAMs MR	[103]

AMPKα1, AMP-activated protein kinase; CCL2, C–C chemokine ligand 2; CCR2, C–C chemokine receptor type 2; CSF-1, Colony-Stimulating Factor 1; CSF-1R, colony-stimulating factor 1 receptor; CXCL12, C–X–C motif chemokine 12; CXCR4, C-X-C chemokine receptor type 4; CpG-ODN, unmethylated cytosineguanine (CpG) oligodeoxynucleotides; IFN-γ, interferon gamma; MARCO, macrophage receptor with collagenous structure; MR, mannose receptor/CD206; NF-κB, nuclear factor kappa B; PI3Kγ, phosphoinositide 3-kinase; STAT, signal transducer and activator of transcription.
